# Prevalence and Evolution of Thyroid Dysfunction in COVID-19: A Retrospective Study

**DOI:** 10.7759/cureus.93542

**Published:** 2025-09-30

**Authors:** Shourya Tadisina, Sobrina Mohammed, Reda Asad, Tatiana Tselovalnikova, Bong Nguyen, Phani V Akella, Maha Abu Kishk

**Affiliations:** 1 Endocrinology, Diabetes and Metabolism, University of Missouri - Kansas City, Kansas City, USA; 2 Biomedical and Health Informatics, University of Missouri - Kansas City, Kansas City, USA; 3 Infectious Diseases, The University of Kansas Health System, Kansas City, USA

**Keywords:** covid-19, hyperthyroidism, hypothyroidism, non-thyroidal illness, thyroid dysfunction

## Abstract

Objective

Coronavirus-19 (COVID-19) is known to mainly affect the respiratory system, but it has also been found to impact multiple endocrine systems. Various studies have shown a relationship between thyroid dysfunction and COVID-19 infection. However, there is controversy around thyroid-inflammatory autoimmune conditions contributing to a worse prognosis of COVID-19 infection. The main objective of our single-center retrospective study is to evaluate the prevalence and evolution of thyroid dysfunction in patients with COVID-19 infection.

Methods

A total of 615 adults with confirmed COVID-19 infection, between March 2020 and December 2023, who had thyroid function tests (TFTs), were included in the study. Patients with pre-existing thyroid disease or on medications affecting thyroid function were excluded. Thyroid dysfunction was defined as any abnormality in TFTs. Statistical analyses were performed using IBM SPSS Statistics for Windows, Version 26 (Released 2018; IBM Corp., Armonk, NY, USA). Differences in continuous variables were assessed using independent sample t-tests, and Pearson’s Chi-square tests were used to compare categorical variables.

Results

The study showed that 84 patients (13.6%) had thyroid dysfunction. The most common abnormalities were non-thyroidal illness syndrome (NTIS, n = 39; 46.4%) and subclinical hypothyroidism (n = 37; 44%), followed by overt hyperthyroidism (n = 6; 7.1%) and overt hypothyroidism (n = 2; 2.3%). The evolution of thyroid dysfunction was followed for about one year by chart review and showed no progression to overt thyroid dysfunction.

Conclusion

We noted that thyroid dysfunction is not uncommon in patients with COVID-19, with subclinical hypothyroidism and NTIS being the most prevalent findings. These abnormalities are often transient and resolve without progression in most cases. The pathogenesis appears to involve both direct viral effects and systemic inflammatory responses. Given the potential overlap between thyroid dysfunction and long COVID symptoms, selective monitoring of thyroid function is warranted in symptomatic individuals.

## Introduction

Coronavirus disease 2019 (COVID-19), caused by the severe acute respiratory syndrome coronavirus 2 (SARS-CoV-2), rapidly developed into a global pandemic, leading to unprecedented public health challenges [1]. The virus primarily targets the respiratory system but can affect multiple organ systems, with manifestations ranging from asymptomatic infection to fatal respiratory disorders [2]. In elderly patients and individuals with other comorbidities, including diabetes mellitus, cardiovascular disease, and obesity, the virus induced both systemic and pulmonary inflammation. Commonly encountered complications include acute respiratory distress syndrome (ARDS), respiratory failure, heart failure, and acute cardiac injury [3].

Patients with COVID-19 infection are mostly asymptomatic or present with mild, flu-like symptoms, while 14% of cases are severe and 5% of cases are critical [4]. The pathogenesis of COVID-19 involves SARS-CoV-2 entering the lungs through the respiratory system and depositing in the lung parenchyma. Thereafter, the spike protein of the virus binds as a receptor to the angiotensin-converting enzyme 2 (ACE2) on the surface of pneumocytes and facilitates its entry into host cells [5,6]. SARS-CoV-2 has a stronger binding affinity to ACE2 compared to SARS-CoV-1, which explains the greater number of infections caused by SARS-CoV-2. In patients with COVID-19 infection, viral RNA has been detected in other body fluids as well, indicating its interaction with other ACE2-expressing organs, leading to extrapulmonary spread and multiorgan involvement [7].

Apart from the respiratory system, SARS-CoV-2 has been shown to have tissue tropism for the cardiovascular, coagulative, nervous, and gastrointestinal systems [8]. ACE2 is also expressed in multiple endocrine organs, with the highest expression noted in the testis, followed by the thyroid gland, and the lowest in the hypothalamus [9-11]. Thyroid follicular cells have receptors for ACE2 and transmembrane serine protease 2 (TMPRSS2), which facilitate the invasion of SARS-CoV-2, triggering an inflammatory cascade and a hyperreactive immune response, culminating in thyroid dysfunction caused by both autoimmune thyroid disease (AITD) and non-thyroidal illness syndrome (NTIS) [12].

There have been various studies showing a relationship between thyroid dysfunction and COVID-19 infection. However, there is controversy around thyroid inflammatory autoimmune conditions causing a worse prognosis of COVID-19 infection. The main objective of our retrospective study is to evaluate the prevalence and evolution of thyroid dysfunction in patients with COVID-19 infection at our institution.

## Materials and methods

Study design and setting

We conducted a retrospective study at University Health, a tertiary care academic medical center. The study was approved by the Institutional Review Board (IRB), with a waiver of informed consent due to its retrospective nature.

Study population

**Figure 1:**
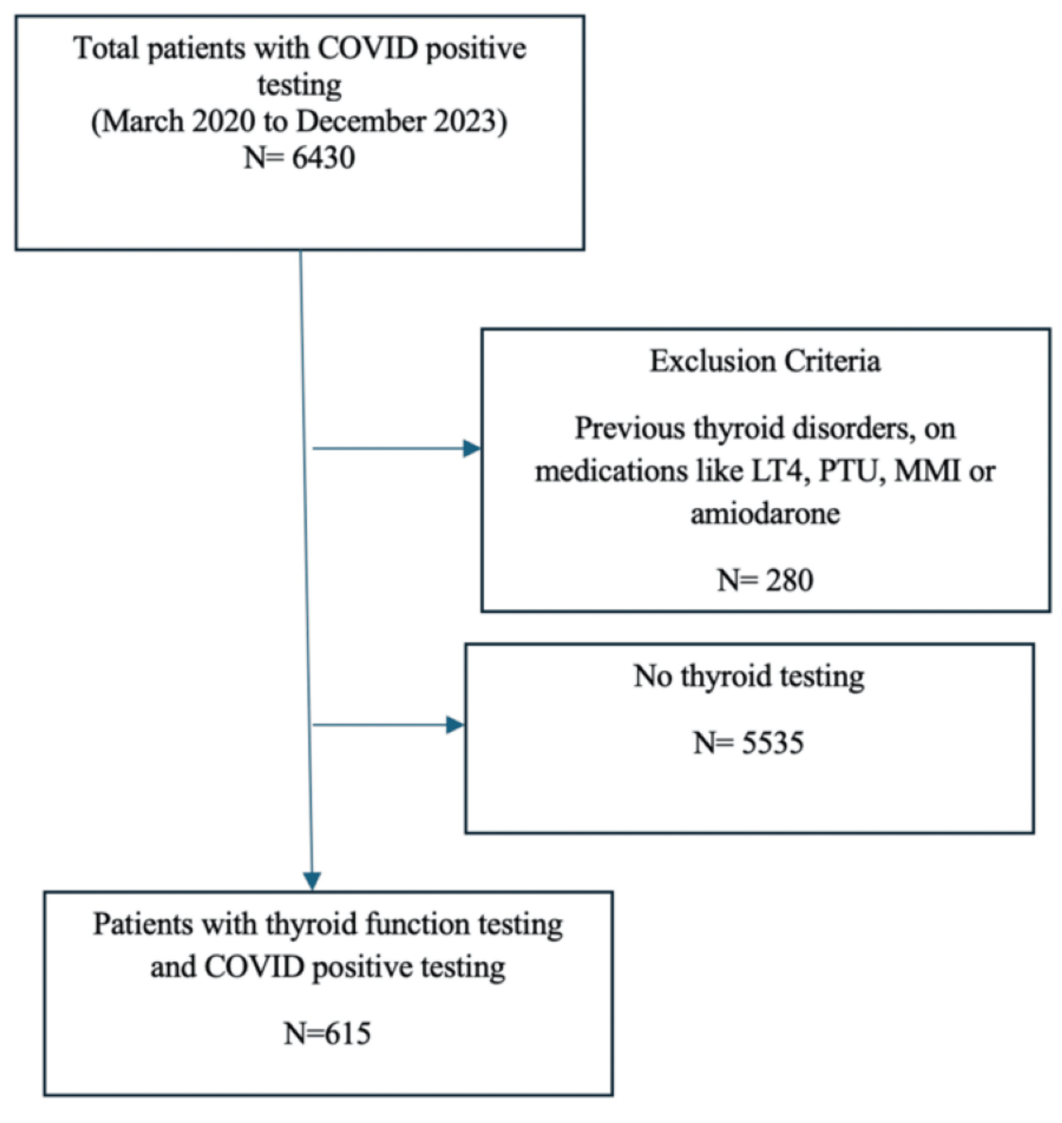
Flow diagram for the study LT4, levothyroxine; MMI, methimazole; PTU, proplythiouracil

Adult patients (age > 18 years; n = 6,430) who tested positive for SARS-CoV-2 by polymerase chain reaction (PCR) between March 2020 and December 2023, in either inpatient or outpatient settings, and had available thyroid function tests (TFTs) during the infection were included in this study. Patients were excluded (n = 280) if they had a known history of thyroid disease, were on thyroid-related medications (levothyroxine, methimazole, or propylthiouracil), or were using medications known to affect thyroid function (e.g., amiodarone or lithium). A total of 615 patients met the criteria and were included in the study (Figure 1).

Data collection and analysis

Data were extracted from the electronic medical record system and included age, sex, ethnicity, body mass index (BMI), clinical setting (inpatient and outpatient), ICU stay, TFTs, COVID-19 PCR, and mortality. TFTs included thyroid-stimulating hormone (TSH) with a normal range of 0.34-5.6 uIU/mL, free thyroxine (FT4) with a normal range of 0.60-1.60 ng/dL, and total triiodothyronine (TT3) with a normal range of 0.60-1.80 ng/dL. Statistical analyses were performed with IBM SPSS Statistics for Windows, Version 26 (Released 2018; IBM Corp., Armonk, NY, USA). Patients meeting the inclusion criteria were then divided into two groups: COVID-19 with thyroid dysfunction (n = 84) and COVID-19 without thyroid dysfunction (n = 531). Differences in continuous variables such as age, BMI, and length of hospital stay were assessed using independent sample t-tests. Pearson’s Chi-square tests were employed to compare categorical variables, including gender, race, BMI category, mortality, mechanical ventilation, and ICU admission. A p-value of <0.05 was considered statistically significant.

## Results

Patient characteristics are described in Table 1. Of the 615 patients identified in our study, thyroid dysfunction was seen in 84 patients (13.6%). The most common thyroid dysfunctions noted were subclinical hypothyroidism (n = 37; 44%) and NTIS (n = 39; 46.4%). Two patients were found to have overt hypothyroidism (2.3%), and six patients had overt hyperthyroidism (7.1%). NTIS is defined as individuals with low TSH but normal free T4 and total T3 levels. Results are shown in Table 2.

**Table 1:** Baseline characteristics and outcomes of patients with and without thyroid dysfunction Continuous variables were compared using the independent samples t-test or the Mann-Whitney U test (for non-normally distributed data). Categorical variables were compared using the Chi-square test or Fisher’s exact test, when appropriate. *p < 0.05 indicates statistical significance. Continuous variables: mean ± standard deviation (SD) with range, or median with range, as appropriate; stay duration is expressed in days, reported as median (range); categorical variables are expressed as counts and percentages; Fisher’s exact test used where expected cell counts <5; χ², Chi-square test statistic; t, independent samples t-test statistic; U, Mann-Whitney U test statistic.

Variable		Thyroid Dysfunction (N = 84)	No Thyroid Dysfunction (N = 531)	Test Statistic	p-value
Age, years; mean ± SD (range)		56.3 ± 16.2 (18-91)	56.4 ± 17.7 (15-102)	t = -0.48	0.478
Gender	Female	47 (56.0%)	259 (46.8%)	χ² = 2.47	0.116
	Male	37 (44.0%)	295 (53.2%)		
Race	White patients	36 (42.9%)	237 (42.8%)	χ² = 2.08	0.353
	Black patients	32 (38.1%)	242 (43.7%)		
	Other patients	16 (19.0%)	75 (13.5%)		
BMI, kg/m²; mean ± SD		30.6 ± 11.6	29.3 ± 10.3	t = 1.48	0.140
BMI category	Underweight (<18.5)	4 (4.8%)	36 (6.5%)	χ² = 0.62	0.924
	Normal (18.5-24.9)	24 (28.6%)	157 (28.3%)		
	Overweight (25-29.9)	21 (25.0%)	145 (26.2%)		
	Obesity (≥30)	31 (36.9%)	193 (34.8%)		
Mortality		8 (9.5%)	41 (7.4%)	χ² = 0.46	0.496
Mechanical ventilation		27 (32.1%)	0 (0.0%)	Fisher’s exact	<0.001*
ICU admission		3 (3.6%)	32 (6.0%)	χ² = 0.79	0.373
Hospital stay duration, days; median (range)		10 (0-150)	12 (0-273)	U = 21,854	0.258

**Table 2:** Results of abnormal thyroid function observed at the diagnosis of COVID-19 infection

Thyroid Dysfunction	Number of Patients
Overt hyperthyroidism	6 (7.1%)
Overt hypothyroidism	2 (2.3%)
Subclinical hypothyroidism	37 (44%)
Non-thyroidal illness	39 (46.4%)

NTIS can be associated with critical illness and mechanical ventilation, so we conducted a chart review and excluded individuals with critical illness, those on mechanical ventilation, and those with previously untreated abnormal thyroid dysfunction; a total of 44 patients were identified. A subgroup analysis of these 44 patients showed that most had mild COVID-19 infection and were admitted for other medical conditions. Only one patient was tested in the outpatient setting, with the remainder tested either as inpatients or in the emergency department. Additionally, only one patient had TFTs performed after receiving dexamethasone, revealing subclinical hypothyroidism.

Among the 44 patients with thyroid dysfunction, overt hyperthyroidism from Graves’ disease was seen in one (2.3%) case; overt hypothyroidism in two (4.5%) cases; subclinical hypothyroidism in 27 (61.3%) cases; and NTIS in 14 (32%) patients. Results are shown in Table 3.

**Table 3:** Results of subgroup analysis of 44 patients after excluding individuals with critical illness and untreated thyroid disorders

Thyroid Dysfunction	Number of Patients
Overt hyperthyroidism	1 (2.3%)
Overt hypothyroidism	2 (4.5%)
Subclinical hypothyroidism	27 (61.3%)
Non-thyroidal illness	14 (32%)

We performed a chart review of individuals with thyroid dysfunction and followed their TFTs over one year to identify the evolution. We noted that, among individuals with subclinical hypothyroidism, repeat testing was done in 48% of them; only 7% (2 out of 27) developed overt hypothyroidism, 26% (7 out of 27) became euthyroid, and 15% (4 out of 27) remained subclinical hypothyroid. Among individuals with NTIS, only 29% had repeat TFTs; 21% (3 out of 14) became euthyroid, and 7% (1 out of 14) developed overt hyperthyroidism, which was from a toxic thyroid nodule.

## Discussion

Our retrospective study contributes to the growing body of literature identifying transient thyroid dysfunction as a frequent occurrence in patients with COVID-19. In a cohort of 615 patients, we identified thyroid abnormalities in 13.6% of cases, with subclinical hypothyroidism and non-NTIS being the most prevalent. These findings align with previous reports demonstrating a substantial incidence of thyroid dysfunction during acute SARS-CoV-2 infection. Arora et al. reported NTIS in 58.8% of their critically ill COVID-19 patients [13], while Ahn et al. also found NTIS to be the most common thyroid disorder in a Korean cohort [14].

One of the key strengths of our study lies in the subgroup analysis of 44 patients, where we excluded individuals with mechanical ventilation and untreated pre-existing thyroid disorders. This allowed us to better evaluate the direct or indirect impact of SARS-CoV-2 on thyroid function. Our longitudinal data showed that the majority of thyroid abnormalities, particularly subclinical hypothyroidism and NTIS, were self-limiting. Only 7% of patients with subclinical hypothyroidism progressed to overt disease, while 21% of those with NTIS returned to euthyroid status. These results are consistent with findings by Muller et al., who reported that thyroid function normalized in all patients over a 12-month follow-up and found no increase in thyroid autoimmunity markers [15].

Such transient abnormalities may reflect an adaptive endocrine response to acute illness, rather than a sign of permanent dysfunction. NTIS, in particular, is a well-documented phenomenon in critically ill patients, including those with COVID-19, sepsis, or post-surgical states, and typically resolves without intervention [16].

The mechanisms underlying thyroid dysfunction in COVID-19 appear to be multifactorial. SARS-CoV-2 may directly infect thyroid follicular cells via ACE2 and TMPRSS2 receptors, facilitating viral entry and cytopathic effects [17]. In support of this, Jakovac et al. identified SARS-CoV-2 antigens in thyroid tissue, showing histopathological features of subacute thyroiditis [18]. However, immune-mediated mechanisms may play an even larger role. Elevated cytokines, especially interleukin (IL)-6 and IL-1β, have been implicated in suppressing hypothalamic-pituitary-thyroid (HPT) axis signaling and altering deiodinase activity [19]. Specifically, IL-1β suppresses deiodinase (DIO) 1, reducing peripheral T3 levels, while NF-κB-driven inflammation upregulates DIO2, disrupting local thyroid hormone metabolism [19].

These mechanisms may contribute to NTIS and other thyroid abnormalities. Elevated IL-6 and C-reactive protein (CRP) levels have also been found in patients with post-COVID-19 painless thyroiditis, further supporting a cytokine-driven pathophysiology [20].

Thyroid hormones themselves influence immune function. Hypothyroidism has been associated with impaired immune responses, while hyperthyroidism can exaggerate systemic inflammation [21]. However, the relationship between thyroid status and immune function remains complex, with conflicting findings reported in the literature [21]. In our cohort, the predominance of subclinical hypothyroidism and NTIS, both associated with low or borderline thyroid hormone levels, raises the possibility that such dysfunctions may reflect or influence immune dynamics during SARS-CoV-2 infection. Although our study was not designed to correlate thyroid status with clinical outcomes or immune biomarkers, the transient nature of these dysfunctions, in most cases, suggests they may represent adaptive rather than pathologic immune-endocrine responses.

Moreover, the potential for SARS-CoV-2 to trigger AITDs is increasingly being explored. In a large population-based study, Peng et al. [22] identified an increased risk of Graves’ disease following COVID-19, particularly among individuals aged 18 to 40 years. In our study, we observed one case of overt hyperthyroidism, attributed to a toxic nodule likely unrelated to SARS-CoV-2, underscoring the need for follow-up evaluation to distinguish between transient and autoimmune causes of dysfunction.

Post-COVID-19 syndrome, often referred to as "long COVID," is a condition characterized by fatigue in more than 50% of affected individuals. This non-specific symptom shares similarities with hypothyroid presentations, thereby warranting selective TFT in patients exhibiting persistent symptoms, and has led to the investigation of the relationship between thyroid function and long COVID. Our data support this approach: most cases of subclinical hypothyroidism and NTIS resolved over a 12-month period, but a small subset progressed to overt thyroid disease. Specifically, 7% of patients with subclinical hypothyroidism developed overt hypothyroidism, and 21% of those with NTIS returned to euthyroid states. These observations are consistent with recommendations from Lui et al. [19], who advocate retesting thyroid function approximately six weeks after recovery, especially in those diagnosed with NTIS. Our findings reinforce the clinical utility of post-acute thyroid monitoring, as early detection of persistent or evolving dysfunction may allow for timely intervention in affected individuals.

Our study is not without limitations. The retrospective design introduces potential selection bias, as TFTs were more likely to be performed in symptomatic or hospitalized patients, and only a few patients had total TT3 levels. Furthermore, only a subset of patients underwent repeat TFTs, constraining conclusions regarding long-term outcomes.

Prospective studies with standardized testing protocols and long-term follow-up are necessary to validate our findings. Inclusion of thyroid imaging and antibody panels would help clarify whether SARS-CoV-2 can induce autoimmune thyroiditis or simply unmask pre-existing thyroid predisposition. Research should also assess whether thyroid dysfunction has prognostic value in COVID-19, or if thyroid hormones may serve as therapeutic targets during the acute or recovery phases.

## Conclusions

Our study demonstrates that thyroid dysfunction is not uncommon in patients with COVID-19, with subclinical hypothyroidism and NTIS being the most prevalent findings. Fortunately, these abnormalities are often transient and resolve without progression in most cases. The pathogenesis appears to involve both direct viral effects and systemic inflammatory responses. Given the potential overlap between thyroid dysfunction and long COVID symptoms, selective monitoring of thyroid function is warranted, especially in symptomatic individuals. Further investigation is essential to delineate the prognostic, diagnostic, and therapeutic implications of thyroid involvement in SARS-CoV-2 infection.
